# Correlation of Biometric Parameters With Endothelial Distance in EVO ICL Phakic Lens Implantation

**DOI:** 10.1155/joph/6959314

**Published:** 2025-06-12

**Authors:** Beltrán-Murcia J., Álvarez-Rementería Capelo L., Blázquez-Sánchez V.

**Affiliations:** ^1^Department of Optometry and Vision, Faculty of Optics and Optometry, Universidad Complutense de Madrid, Madrid, Spain; ^2^Clínica Rementería, Madrid, Spain

**Keywords:** endothelium-ICL, EVO ICL, implantable Collamer lens, phakic lens, vault

## Abstract

**Purpose:** The correlations between different parameters and the positioning of the EVO ICL were analyzed. Significant correlations were observed, highlighting the relationship between the endothelium and the anterior surface of the phakic ICL lens. However, it is important to note that GAP is only a postoperative measure and cannot be used to directly define the correct positioning of the EVO ICL. The study emphasizes these correlations, without drawing conclusions regarding the causal relationships between the parameters.

**Methods:** In this retrospective, observational, transversal analytic study, patients who underwent refractive surgery with implanted EVO ICL pIOL were analyzed. The distance between corneal endothelium and anterior surface of ICL (GAP) was measured postoperatively at 1 week and 1 month using ANTERION AS-OCT.

**Results:** 35 eyes of 35 patients were analyzed. The mean GAP values at 1 week and 1 month were 2.5 ± 0.2 mm and 2.6 ± 0.2 mm, respectively (*p* = 0.002), increasing its value. The GAP value correlated positively with preoperative anterior chamber volume, white-to-white, lens vault, spur-spur distance, anterior chamber angle distance, and anterior chamber angles values.

**Conclusions:** The correlations between GAP and pre- and postoperative variables highlight its relationship with anterior segment anatomy after EVO ICL implantation. However, as a postoperative measurement, GAP does not determine the correct positioning of the lens.

## 1. Introduction

Currently, in addition to refractive corneal treatments, the implantation of a phakic intraocular lens (pIOL) is a viable option to treat low to high ametropias [1]. This procedure is safe, effective, reversible, and stable, gaining popularity with the model featuring a central-port design [2–4]. EVO ICL (STAAR Surgical, Monrovia, CA, USA) is a pIOL designed for the implantation in the posterior chamber, positioned in the sulcus, and capable of compensating for myopia, hyperopia, and astigmatism [5]. Made from Collamer, a copolymer of collagen and HEMA, the lens offers excellent biocompatibility [6].

The EVO ICL is available in four sizes for myopic models (12.1, 12.6, 13.2, and 13.7 mm) and four sizes for hyperopic Visian ICL (11.6, 12.1, 12.6, and 13.2 mm) [7]. The manufacturer's OCOS (Online Calculation and Ordering System, STAAR Surgical, Monrovia, CA, USA) recommends the lens power and size based on the preoperative patient's refraction and WTW and ACD values.

Postimplantation, it is crucial to accurately determine the lens position within the eye and its interaction with surrounding structures. The correct lens size is inferred from distances between the pIOL and various anatomical structures. Traditionally, the vault, defined as the distance between the posterior surface of the pIOL and the anterior surface of the crystalline lens, has been used to assess lens sizing. An optimal vault ranges from 250 to 750 μm. However, vault can be influenced by several factors such as pupil dynamics, light conditions, accommodation, age, and the crystalline lens rise (CLR) [8–11], making it a less reliable sole measurement for determining the correct pIOL size.

Studies have shown that iridocorneal angles change after EVO ICL implantation, leading to a reduction in angle values [12–15], which can increase the risk of intraocular pressure, synechiae, or closed-angle glaucoma [12–14, 16]. The correlation between vault and angular closure remains unclear, with some models predicting outcomes based on compression (ICL size-ATA) and preoperative pupil diameter. Thus, we introduce the Glance for Anterior Positioning (GAP) distance, defined as the distance between the corneal endothelium and the anterior surface of the pIOL. This measure could be less influenced by variables affecting vault.

The purpose of this study is to assess the GAP distance in the immediate and medium postoperative periods and to examine its relationships with preoperative and postoperative variables following the implantation of the EVO ICL pIOL.

## 2. Setting

Clínica Rementería, Madrid. Spain.

## 3. Methods

### 3.1. Patients and Inclusion Criteria

This retrospective observational transversal analytic study included patients who had the EVO ICL (STAAR Surgical, Monrovia, CA) (V4c/V5 models) pIOL implanted for myopia correction between 2019 and 2022 at Clínica Rementería, Madrid, Spain. Only eyes of patients who completed postoperative follow-ups were included. Inclusion criteria were age 21–60 years, stable refraction in the last 12 months before surgery, preoperative refraction from −0.50 D to −18.00 D, anterior chamber depth (ACD) ≥ 2.80 mm, endothelial cell density > 2200 cells/mm^2^, IOP 10–21 mmHg, and absence of pathologies preventing surgery. Exclusion criteria included patients not meeting inclusion criteria, amblyopic and/or single eyes, postsurgical complications, previous ocular or systemic diseases affecting surgery or outcome, vertically implanted lenses, hypermetropic patients, and those not adhering to postoperative visit schedules.

### 3.2. Study Parameters

Subjective refraction was performed by an experienced optometrist under physiological and cycloplegic conditions at 12 mm from the corneal vertex. UDVA and CDVA were measured using a Bailey–Lovie optotype at 4 m. Preoperative refraction was based on autorefractometry with and without cycloplegia.

ANTERION was used for preoperative and postoperative measurements. This swept-source OCT biometer, operating at a wavelength of 1300 nm, provides detailed tissue imaging with axial resolution < 10 μm. It captures aqueous depth (AQD), central cornea thickness (CCT), lens thickness (LT), and white-to-white (WTW) values.

Preoperative measurements included AQD ( = ACD), CCT, anterior chamber volume (ACV), WTW, LT, lens vault (LV), pupil diameter, spur-spur (SS-SS) distance, ACA distance, and angular measurements ACA_500_N, ACA_500_T, AOD_500_N, AOD_500_T, TISA_500_N, and TISA_500_T. Postoperative measurements of GAP and vault were taken at 1 week and 1 month. Angular measurements and SS-SS distance were also recorded for the same period.

All visual acuity tests and measurements were taken under controlled luminance conditions: 262 lux for the refraction room and 45 lux for the ANTERION scanning room, using an IsoTech 1335 Luxometer (TES Electrical Electronic Corp., Taipei, Taiwan).

### 3.3. EVO ICL Size and Power Calculation

Using its modified vertex formula, OCOS was used to calculate the pIOL power and size based on WTW and ACD values from Pentacam HR (Oculus, Wetzlar, Germany). Measurements were obtained in the horizontal meridian.

### 3.4. Surgical Technique

The surgical implantation of the EVO ICL involved dilating the pupils 30 min prior to surgery. All procedures were performed under topical anesthesia. Provisc viscoelastic material was injected into the anterior chamber to protect the ocular structures, and the EVO ICL lens was inserted through a 3.2 mm corneal incision. The viscoelastic material was removed using CENTURION Vision System (Alcon Surgical), and all incisions were closed with stromal hydration. Postsurgical care included topical antibiotics and steroids.

### 3.5. Statistics

The sample size for the study was calculated using GRANMO v.7.12 (Institut Municipal d'Investigació Mèdica, Barcelona, Spain). The primary study variable was the mean and standard deviation of ACA500 at 180° (iridocorneal angle measured at 500 μm from the scleral spur). Paired means were employed to assess differences between preoperative and postoperative measurements. The error considerations included an alpha risk (α) of 0.05, representing a 5% probability of a Type I error, and a beta risk (β) of 0.20, corresponding to a statistical power of 80%, ensuring the probability of detecting real differences if they exist. The estimated variability accounted for a standard deviation of the differences of 10° and a minimum detectable difference of 5°. Although the study was retrospective and no follow-up losses were reported, a loss rate of 0.001% was considered in the sample size calculation. Based on these parameters, GRANMO determined that at least 32 eyes were needed to achieve adequate statistical power, with an additional percentage included to compensate for possible loss of subjects during the study.

All data were collected in a worksheet. For the statistical analysis, all data were processed and analyzed using IBM SPSS Statistics V25 Software (IBM, Armonk, New York, USA). Shapiro–Wilk, t-Student, and Wilcoxon tests were performed to confirm data normal distribution. Pearson and rho-Spearman correlations were also used to confirm statistic relationship among variables (*p* < 0.05).

## 4. Results

The study included 35 right eyes of 35 patients (51.4% women) implanted with EVO ICL for ametropia correction. Demographic and preoperative values from Pentacam are shown in Table 1. Preoperative values from ANTERION are given in Table 2.

The GAP distance was 2.5 ± 0.2 (range 2.11–3.05) mm at 1 week and 2.6 ± 0.2 (range 2.2–3.1) mm at 1 month, representing a mean of 0.04 ± 0.08 mm (*p* = 0.002). Vault was 467 ± 189 (range 109–882) μm at 1 week and 429 ± 171 (range 42–732) μm at one month, and a decrease of −38 ± 79 μm (*p* = 0.008) was found.

Preoperative ACA_500_N was 59 ± 12 (range 38–76)°, decreasing to 36 ± 7 (range 21–53)° at 1 week and 35 ± 8 (range 19–61)° at 1 month. A mean decrease of −22 ± 9° (*p* < 0.001) and −23 ± 9° (*p* < 0.001) at 1 week and 1 month, respectively, was found. No changes were found between one week and one month postoperatively (*p* = 0.393). Preoperative ACA_500_T was 55 ± 12 (range 33–73)° decreasing to 33 ± 8 (range 21–53)° at 1 week after surgery and 32 ± 8 (range 18–53)° at 1 month, and a mean decrease of −22 ± 11° (*p* < 0.001) at 1 week and −23 ± 10° (*p* < 0.001) at 1 month was found. Changes in postoperative period were not statistically significant (*p* = 0.397).

SS-SS measurement increased from 12 ± 0.5 (range 11–13) mm preoperatively to 12 ± 0.5 mm (range 11–13) (*p* < 0.001) at 1 week and 12 ± 0.5 (range 11–13) mm at 1 month (*p* < 0.001). Variation between week and month remained constant (*p* = 0.7).

Compression defined as ICL Size-SS-SS distance was 0.9 ± 0.04 (range 0.1–1.8) mm preoperatively, decreasing to 0.8 ± 0.4 (range 0.07–1.59) mm at 1 week (*p* < 0.001) and 0.8 ± 0.4 mm (range 0.1–1.6) at 1 month (*p* < 0.001). ICL compression did not change during postop follow-up (*p* = 0.7).

All the variables except for GAP and vault did not show statistically significant changes between 1 week and 1 month after surgery. All statistical treatment is performed with the postoperative values at one month follow-up.

**Figure 1:**
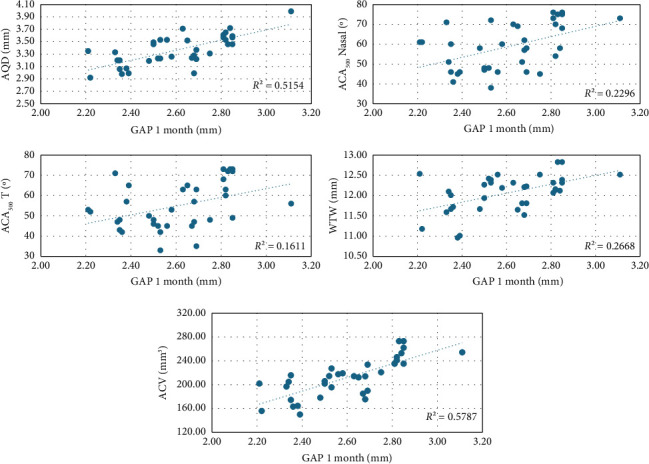
Bivariate correlations of 1-month GAP with preoperative AQD, ACA_500_N, ACA_500_T, WTW, and ACV values.

Bivariate correlations of GAP value at 1 month with the preoperative variables are shown in Table 3. Correlations of GAP at 1 month with some preoperative variables can be seen in Figure 1.

No statistically significant correlation was found between GAP value at 1 month and the preoperative values of cylinder (*R* = 0.04, *p* = 0.8), age (*R* = 0.06, *p* = 0.7), CCT (*R* = 0.08, *p* = 0.6), LT (*R* = −0.2, *p* = 0.4), pupillary diameter (*R* = 0.3, *p* = 0.06), and compression value (*R* = −0.2, *p* = 0.3).

The correlations of vault at 1 month with the preoperative variables were also studied. Statistical significance was found with AQD (*R* = 0.5, *p* < 0.001), LT (*R* = −0.5, *p* = 0.003), and LV (*R* = −0.5, *p* < 0.001). No statistical significance was found with the rest of the variables.

**Figure 2:**
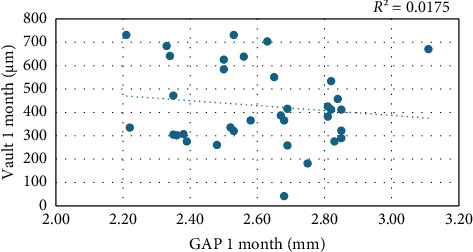
Bivariate correlation between the value of GAP 1-month and vault 1-month values.

Bivariate correlation between GAP and vault at 1 month was poor and without statistical significance (*R* = −0.1, *p* = 0.4), indicating that variation in GAP does not lead to variation in vault and vice versa (Figure 2).

Postoperatively, GAP at 1 month correlates with the values at 1 month of ACA_500_N (*R* = 0.4, *p* = 0.03), ACA_500_T (*R* = 0.3, *p* = 0.05), AOD_500_N (*R* = 0.5, *p* = 0.004), AOD_500_T (*R* = 0.5, *p* = 0.004), TISA_500_N (*R* = 0.4, *p* = 0.01), and TISA_500_T (*R* = 0.4, *p* = 0.007). The one-month vault showed no statistically significant correlation with ACA_500_N is (*R* = 0.03 *p* = 0.9) or with ACA_500_T is (*R* = −0.2 *p* = 0.3).

The linear equations describing the correlations between the value of GAP at 1 month and the value of ACA_500_N and ACA_500_T at 1 month are:

(1) $$\begin{array}{c} \text{ACA}500N1\text{Month}=-1.6+\left(14.3\times \text{GAP}1\text{Month}\right),\\ \text{ACA}500T1\text{Month}=-0.4+\left(13\times \text{GAP}1\text{Month}\right). \end{array}$$

These equations indicate that for 1 mm of GAP variation, ACA_500_N could vary by up to 14° on average and ACA_500_T would vary by up to 12° on average.

GAP value does not correlate statistically with the angular closures of ΔACA_500_N (*R* = −0.3 *p* = 0.09) or ΔACA_500_T (*R* = −0.2, *p* = 0.3), although a negative trend in their relationship can be seen. Dividing the sample according to the mean GAP value at 1 month and the mean variation of ACA_500_N and ACA_500_T, the distribution of the sample is shown in Table 4. Higher GAP values tend to have higher ACA_500_N and ACA_500_T angular variations compared to those with GAP less than the mean.

**Figure 3:**
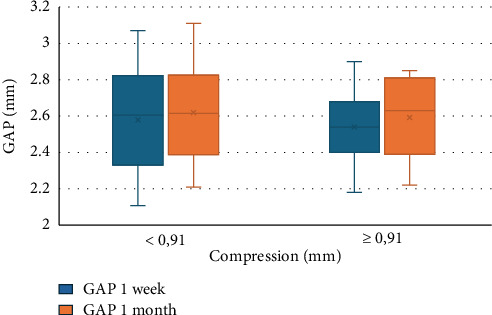
1-week and 1-month vault values divided by mean compression.

**Figure 4:**
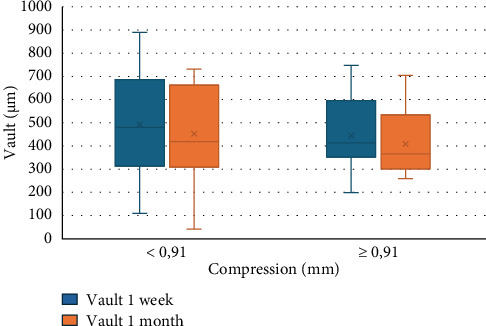
1-week and 1-month GAP values divided by mean compression.

Although no correlation is found between GAP and compression, in Figure 3, it is shown how the sample is divided based on mean compression according to GAP values at 1 week and 1 month. Similarly, Figure 4 shows the distribution of vault according to mean compression at 1 week and 1 month.

From the preoperative data and using stepwise linear regression calculations, we obtained the GAP prediction formula.

**Figure 5:**
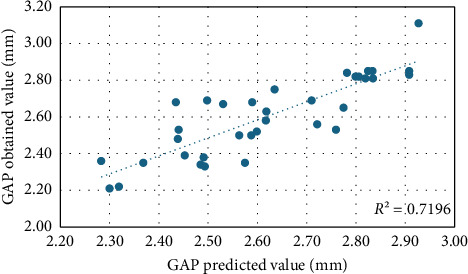
Distribution of GAP prediction versus GAP obtained values at 1-month postop.

Equation 1. Formula predicting GAP distance between the corneal endothelium and the anterior surface of the EVO ICL phakic lens.

(2) $$\begin{array}{c} \text{GAP}=3.304+\left(0.07\times \text{ACV}\right)+\left(0.23\times \text{SphPreop}\right)+\left(-0.168\times \text{SS}-\text{SS}\right), \end{array}$$

where ACV relates to preoperative sphere and the SS-SS distance, obtaining an *R*^2^ = 0.7 for our data sample (Figure 5).

## 5. Discussion

The implantation of the EVO ICL phakic lens is an increasingly popular technique for correcting ocular ametropia. The success of this procedure depends, among other factors, on the correct selection of lens size.

Vault has been one of the most important values to determinate the correct sizing and positioning of the ICL lens once implanted. In this study, a mean decrease in vault value between 1 week and 1 month of follow-up of −38 ± 79 μm (*p* = 0.008) was found. Other studies also report this decrease in vault value over time (236–238). Alfonso et al. [17] observed a variation of −71 ± 58 μm during the first 6 months of follow-up and Fernandez-Vega-Cueto et al. [18] reported a mean vault value of 409 ± 196 μm at 12 months, decreasing to 357 ± 178 µm at 24 months. However, vault is a variable dependent on pupil dynamics, light conditions, accommodation, and CLR value, as described by Gonzalez-Lopez et al. [10, 19], who noted a mean variation of 167 ± 70 μm in vault due to pupil changes between photopic and mesopic conditions. Furthermore, Gonzalez-Lopez et al. observed significant differences in mean vault values in patient groups with CLR values less than 0 μm and greater than 350 μm, demonstrating that high vault values may correspond to posteriorized or less curved crystalline lenses. Therefore, vault alone cannot be considered the sole determinant of whether a lens size is suitable for a particular anatomy.

In this study, the variable GAP is defined as the distance between the corneal endothelium and the anterior surface of the phakic lens. In our study, GAP distance increased from 2.5 ± 0.2 mm (range 2.11–3.05) to 2.6 ± 0.2 mm (range 2.21–3.11) at 1 week and 1 month of follow-up, respectively, representing a mean increase of 40 μm. This change was statistically significant (*p* = 0.002). In contrast, vault, the distance between the posterior surface of the lens and the anterior surface of the crystalline lens, showed a mean decrease of −37.71 ± 78.98 μm between one week and one month postoperatively, also reaching statistical significance (*p* = 0.008). Vault reduction over time has been reported in other studies and may influence lens stability and corneal health. Meanwhile, the increase in GAP suggests greater separation between the lens and the endothelium, potentially reducing the risk of endothelial damage. However, the specific clinical implications of this change remain to be fully determined.

No statistically significant correlation was found between GAP and vault (*R* = −0.132, *p* = 0.448), indicating that these values may vary independently. However, a positive correlation was observed between GAP and several postoperative angular structures, suggesting that a higher GAP tends to be associated with larger iridocorneal angles and a lower risk of angle closure. Conversely, a reduced GAP could imply a higher risk of angle closure, although no severe angle closure cases were documented in the analyzed sample. Calvo-Sanz et al. [20] measured this distance using Visante AS-OCT obtaining a value of 2.3 ± 0.08 mm during the first month of follow-up, which fits with our results. Yang et al. [21] studied the C-ICL distance (endothelium-anterior ICL) with Pentacam and described values of 2428 ± 248 μm and 2425 ± 246 μm at 3 months and 4 years after surgery, respectively, although no statistically significant relationship between the two values was found. Le Loir and Cochener [22] described the E-ICL distance and measured it with Visante AS-OCT obtaining a mean value of 2.4 ± 0.4 mm at 3 months, which remained stable during the 60-month follow-up. For this sample, Le Loir used the V4b model without central port. The central port in the V4c EVO ICL models improves aqueous humor dynamics with a bellows effect on the lens due to pupil interaction; however, the V4b models, although they may also show movement due to pupillary dynamics, would not have this bellows effect, which could keep the GAP distance measurements stable at follow-up.

On the other hand, Cao et al. [23] found that in patients implanted with V4b Visian ICL model, the mean GAP distance increased between 1 and 3 months of follow-up from 2230 ± 253 μm to 2266 ± 269 μm respectively, although without a statistically significant difference. After 3–24 months follow-up, they described an increasing trend in GAP distance, but no statistically significant difference during follow-up was found. Measuring GAP distance with BMU and 6-month follow-up, Elshafei et al. [24] found the GAP distance to be 2826 ± 331 μm, again with V4 models without a central port. Pitault et al. [25] also measured GAP in a sample of 14 patients implanted with a V4 model and an average follow-up of 13 months, obtaining a value of 2398 ± 203 μm. Zhang et al. went a step further by measuring and comparing GAP distance between two devices in a group of patients implanted with V4 ICL model and a mean follow-up of 19 months. They found a mean GAP value measured with Visante AS-OCT of 2.5 ± 0.3 mm and 2.5 ± 0.3 mm measured with UBM although these were not statistically significant (*p* > 0.005).

However, none of these studies have explored the relationships that GAP distance may have with preoperative and postoperative parameters. The aim of this study to define the relationships that GAP distance has with pre- and postoperative variables.

In our sample, we found that GAP correlated positively with preoperative AQD, ACV, WTW, SS-SS, ACA distance, preoperative sphere, EVO ICL size, and EVO ICL power and negatively with LV (Table 3). Eyes with larger anterior segment anatomies, deeper chambers, larger ACVs, more posteriorized crystalline lenses, WTW, or larger SS-SS and ACA distances tended to have higher postoperative GAP values. Changes in GAP and vault are statistically significant; however, their clinical impact depends on the specific context. While vault reduction may affect lens stability, an increase in GAP appears to be associated with improved angular conditions and a lower risk of closure. Nevertheless, further studies are needed to establish a clear link between GAP and patient-reported complications.

It was also found that GAP had a statistically significant correlation with preoperative angular variables ACA_500_N, ACA_500_T, AOD_500_N, AOD_500_T, TISA_500_N, TISA_500_T, SSA_500_N, and SSA_500_T (Table 3). This indicates that angular structures and wide or open preoperative angles will tend to have higher GAP values, with the phakic lens being more separated from the endothelium.

On the other hand, vault correlates only with preoperative measures of AQD, LT, and LV, while it does not correlate with any of the other preoperative variables measured, preoperative angular structures, or compression values. This might explain why it is difficult to predict postoperative vault values from preoperative variables. Numerous authors have developed formulas for predicting vault from preoperative biometric data, but the reliability of these formulas is not optimal [26–29]. This may be because the samples of eyes used for formula development and analysis are small and, as we found in our sample, the vault value correlates poorly with preoperative variables, complicating its prediction.

With a focus on the postoperative phase, our findings suggest that the GAP value correlates with the one-month values of ACA_500_N, ACA_500_T, AOD_500_N, AOD_500_T, TISA_500_N, and TISA_500_T. In our sample, eyes with larger GAP distances tended to have larger postoperative angular angles and structures. However, vault does not correlate significantly with the angular values at 1 month ACA_500_N, ACA_500_T, AOD_500_N, AOD_500_T, TISA_500_N, and TISA_500_T. Obtaining more closed angular structures is much more correlated with the GAP than it is with vault. This could confirm that a high vault value should not be associated with angular closure, but a decreased GAP value may be associated with closure of angular structures. Furthermore, this fact is confirmed by the poor correlation between the 1-month GAP and vault values (*p* = 0.448), indicating that these values are not related: having a decreased GAP value does not imply a high vault, i.e., we could have a high vault with a high GAP value, which would correspond to a case with a very negative LV, without angular closure.

The variation of the angular structures ΔACA_500_N and ΔACA_500_T between preoperative and 1 week and 1 month follow-up is shown in Table 5, with a decrease of −23±9° (*p* < 0.001) and −23 ± 10° (*p* < 0.001), respectively, at 1 month after surgery. No correlation between GAP and the variation in ΔACA_500_N (*R* = −0.3, *p* = 0.09) and ΔACA_500_T (*R* = −0.2, *p* = 0.3) values was found, although the negative trend could indicate that higher GAP values would be associated with greater angular variations. In fact, dividing the sample according to the mean GAP value and the mean variation of ACA_500_N and ACA_500_T (Table 4), it is observed that when the GAP distance is higher than the mean, there is a greater number of cases with angular variations of ACA_500_N and ACA_500_T higher than the mean. Conversely, when GAP distance is below the mean, most of the angular variations of ACA_500_N and ACA_500_T are below the mean. The fact that no statistically significant correlation was found could be due to the small sample size, and, on the other hand, to the fact that there were no cases with a high angular closure.

When studying the compression, the result was 0.9 ± 0.04 (range 0.09–1.8) mm. When measuring the SS-SS distance, it was found to increase from 12 ± 0.5 mm preoperatively to 12 ± 0.5 mm (*p* < 0.001) at 1 week and 12 ± 0.5 mm (*p* < 0.001) at 1 month after surgery. This change in the SS-SS distance measurement could be due to the thrust that the lens generates in the sulcus which could be transferred to the anterior chamber, causing this minimal variation in the distance from SS-SS. This change in the SS-SS distance causes compression to decrease at 1 month postoperatively to a value of 0.8 ± 0.4 mm, with a statistically significant difference from the preoperative value (*p* < 0.001).

No statistical correlation was found between GAP and compression (*R* = −0.2, *p* = 0.3); however, there is a statistically significant relationship with the values from which compression is derived, i.e., with the SS-SS value (*p* = 0.008) and ICL size (*p* = 0.05). This may be due to the fact that the size selection in this study tends to decrease the final implanted size (Table 1) in 17% of cases depending on the surgeon's experience and preoperative variables, which implies a decrease in the compression suffered by the lens. Figure 3 shows how cases with less than average compression would tend to have a constant GAP between one week and one month of follow-up, while GAP distance increases during follow-up in cases with greater than average compression. This may be due to the change in the SS-SS distance and similarly the compression at 1 month after surgery, causing compression to decrease, and therefore GAP distance may increase. Something similar happens with the vault values if we divide them according to the compression value (Figure 4). Vault values at 1 week tend to be higher in cases of lower-than-mean compression, and vice versa. Higher vault values are observed in cases of lower compression at 1 month. The opposite would be expected: greater vault when compression is greater, as described by other authors [12, 29, 30]. In these cases, the impact of the CLR and the role of the iris sphincter exerting pressure could explain these results.

The strong relationships that GAP has with preoperative anatomical values are also evident in the prediction formula. Relating the ACV, along with the SS-SS distance and the preoperative sphere, a reliability of 72% is achieved, even though the sample of eyes is small.

Nishida et al. [31] also studied GAP distance in a sample of 116 eyes with AS-OCT CASIA2, indicating that post-ACD distance (GAP in our case) measured from endothelium to anterior face of phakic lens had a value of 2.4 ± 0.3 mm at 3 months. In our case, the GAP value at 1 month is higher, 2.6 ± 0.2 mm; this difference could be because the Asian eyes analyzed by Nishida et al. are anatomically smaller than Caucasian eyes, although the biometric parameters of the sample, beyond the ACD value, are not specified in the study. In his study, a formula for predicting post-ACD distance was developed, which includes as predictor variables ACD, ICL size, ATA distance, iris curvature, LV, and TISA_250_, obtaining an *R*^2^ = 0.661. In our case, the linear regression formula includes the variables ACV, SS-SS distance, and preoperative sphere value. The author found, as in our results, a positive correlation between the preoperative ACD and ATA and the post-ACD value, indicating that higher anterior chamber values will result in lenses that are more distant from the endothelium.

This study has some limitations. Firstly, its retrospective design inherently carries biases that could affect the generalizability of the results. In addition, the small sample size limits the statistical power and prevents subgroup analyses that could enrich the understanding of the GAP behavior under different anatomical conditions. A larger sample would allow stratification by ICL sizes and preoperative ACD, particularly to assess GAP correlations in eyes with ACD above or below 3.00 mm. These subgroup analyses would provide more robust evidence to support the clinical applicability of the GAP parameter and facilitate its integration into lens sizing algorithms. Another limitation is the short follow-up period; long-term data would be desirable to evaluate the stability of the GAP over time. Furthermore, the largest ICL size (13.7 mm) was not used in this cohort, due to a surgical tendency to favor smaller lens sizes in order to reduce the risk of angle closure, which also explains the predominance of low vault values observed. Finally, Equation 1, developed from this sample, needs to be validated with an independent cohort before it can be reliably applied in clinical practice.

## 6. Conclusion

GAP distance between the corneal endothelium and the anterior surface of the EVO ICL phakic lens increases during the first postoperative month. It has a very high correlation with many preoperative variables, making it easily predictable from regression formulas. Similarly, there is a strong correlation of GAP with pre- and postoperative angular values.

These relationships mean that the GAP measurement is a variable that can be considered a good indicator of the correct positioning of EVO ICL lenses after implantation.

## Figures and Tables

**Table 1:** Descriptive statistics of the sample.

***N* = 35**	

M/F (%)	49/51
Right eye (%)	100

**Variable**	**Mean ± SD (min, max)**

Age (y)	32 ± 4.5 (23, 41)
Preop Sph (D)	−7 ± 3 (−15, −1.00)
Preop Cyl (D)	−0.7 ± 0.7 (−2.5, 0.00)
WTW (mm)^∗^	12 ± 0.4 (10.8, 12.6)
ACD (mm)^∗^	3.32 ± 0.25 (3, 4)
Iridocorneal angle (°)^∗^	43 ± 5 (33, 57)
K1 (D)^∗^	44 ± 2 (40, 47)
K2 (D)^∗^	45 ± 2 (41, 48)
EVO ICL power SE^†^ (D)	−9±3 (−16, −2)
EVO ICL model	EVO ICL: 9%
	EVO + ICL: 91%
Calculated size^†^ (mm)	12.1: 3/9%
	12.6: 5/14%
	13.2: 27/77%
Implanted size (mm)	12.1: 6/17%
	12.6: 8/23%
	13.2: 21/60%

^∗^Data extracted from Pentacam HR.

^†^Data calculated with OCOS.

**Table 2:** Descriptive statistics of data extracted with ANTERION AS-OCT.

ANTERION	
*N* = 35	Mean ± SD (min, max)
WTW (mm)	12.0 ± 0.5 (11, 13)
AQD (mm)	3 ± 0.2 (3, 4)
CCT (mm)	0.5 ± 0.03 (0.5, 0.6)
ACA_500_N (°)	59 ± 12 (38, 76)
ACA_500_T (°)	55 ± 12 (33, 73)
AOD_500_N (mm)	0.8 ± 0.3 (0.4, 1.4)
AOD_500_T (mm)	0.9 ± 0.3 (0.5, 1.4)
TISA_500_N (mm^2^)	0.3 ± 0.1 (0.1, 0.5)
TISA_500_T (mm^2^)	0.3 ± 0.1 (0.1, 0.6)
SSA_500_N (°)	59 ± 9 (41, 75)
SSA_500_T (°)	59 ± 9 (43, 74)
ACV (mm^3^)	212 ± 32. (150, 273)
ACA (ATA) (mm)	12 ± 0.5 (11, 13)
SS-SS (mm)	12 ± 0.5 (11, 13)
LT (mm)	4 ± 0.2 (3, 4)
LV (mm)	−0.1 ± 0.2 (−0.5, 0.2)
Pupil Ø (mm)	5 ± 1 (4, 7)

**Table 3:** Bivariate correlations of GAP 1 month with the preoperative variables studied.

	GAP 1 month	
	*R*	*p*
Preop Sph	0.3	0.04
AQD	0.7	< 0.001
ACV	0.8	< 0.001
WTW	0.5	0.001
LV	−0.4	0.04
SS-SS	0.4	0.008
ACA	0.5	0.005
ACA_500_N	0.5	0.006
ACA_500_T	0.4	0.010
AOD_500_N	0.6	< 0.001
AOD_500_T	0.6	< 0.001
TISA_500_N	0.5	0.002
TISA_500_T	0.5	0.002
SSA_500_N	0.6	< 0.001
SSA_500_T	0.5	0.004
Preop Cyl	0.04	0.8
Age	0.06	0.7
CCT	0.09	0.6
LT	−0.2	0.4
Pupil Ø	0.3	0.06
EVO ICL size	0.3	0.05
EVO ICL power	0.4	0.02

**Table 4:** Distribution of *the* sample according to the mean values of GAP 1-month and Δ ACA_500_N and Δ ACA_500_T.

		GAP 1 month	
		≤ 2.60	> 2.60
Δ ACA_500_N	> 23.20°	6	13
	≤ 23.20°	11	6

Δ ACA_500_T	> 23.20°	4	8
	≤ 23.20°	13	10

**Table 5:** Preoperative, 1-week, and 1-month measurements and their differences for GAP, vault, ACA500N, ACA500T, pupillary diameter, SS-SS distance, and compression.

Variable	Preop (mean ± sd) (min, max)	1 week (mean ± sd) (min, max)	Dif 1 W-pre (*p* value)	1 Month (mean ± sd) (min, max)	Dif 1M-pre (*p* value)	Dif 1M-1W (*p* value)
GAP (mm)	—	2.5 ± 0.2 (2, 3)	—	2.6 ± 0.2 (2, 3)	—	0.04 ± 0.08 *p* = 0.002
Vault (μm)	—	467 ± 189 (109, 882)	—	429 ± 171 (42, 732)	—	38 ± 79 *p* = 0.08
ACA_500_N (^o^)	59 ± 12 (38, 76)	36 ± 7 (21, 53)	−22.4 ± 9 *p* < 0.001	36 ± 8 (19, 61)	−23 ± 89 *p* < 0.001	−0.8 ± 5 *p* = 0.4
ACA_500_T (^o^)	55 ± 12 (33, 73)	33 ± 8 (21, 53)	−22 ± 11 *p* < 0.001	32 ± 8 (18, 53)	−22.7 ± 10 *p* < 0.001	−0.6 ± 4 *p* = 0.4
AOD_500_N (mm)	0.8 ± 0.3 (0.4, 1.4)	0.4 ± 0.1 (0.2, 0.6)	−0.4 ± 0.2 *p* < 0.001	0.4 ± 0.1 (0.9, 0.6)	−0.4 ± 0.2 *p* < 0.001	−0.02 ± 0.05 *p* = 0.05
AOD_500_T (mm)	0.9 ± 0.3 (0.5, 1.4)	0.4 ± 0.1 (0.2, 0.7)	−0.5 ± 0.3 *p* < 0.001	0.4 ± 0.1 (0.2, 0.7)	−0.5 ± 0.2 *p* < 0.001	−0.01 ± 0.07 *p* = 0.4
TISA_500_N (mm^2^)	0.3 ± 0.1 (0.1, 0.5)	0.15 ± 0.05 (0.04, 0.3)	−0.2 ± 0.08 *p* < 0.001	0.2 ± 0.05 (0.05, 0.2)	−0.2 ± 0.07 *p* < 0.001	−0.01 ± 0.02 *p* = 0.2
TISA_500_T (mm^2^)	0.3 ± 0.1 (0.1, 0.6)	0.14 ± 0.05 (0.04, 0.3)	−0.2 ± 0.10 *p* < 0.001	0.1 ± 0.05 (0.05, 0.3)	−0.2 ± 0.09 *p* < 0.001	−0.01 ± 0.03 *p* = 0.09
Pupil Ø (mm)	5.4 ± 1.00 (3.6, 7.2)	5 ± 0.7 (4, 7)	−0.5 ± 0.9 *p* = 0.003	5 ± 0.9 (4, 7)	−0.4 ± 1 *p* = 0.02	0.2 ± 0.8 *p* = 0.3
SS-SS (mm)	12 ± 0.5 (11, 13)	12 ± 0.5 (11, 13)	0.06 ± 0.08 *p* < 0.001	12.±0.5 (11, 13)	0.06 ± 0.09 *p* < 0.001	0.00 ± 0.04 *p* = 0.7
Compression	1 ± 0.0.4 (0.1, 1.8)	0.9 ± 0.36 (0.07, 1.6)	−0.06 ± 0.08 *p* < 0.001	0.8 ± 0.37 (0.1, 1.6)	−0.06 ± 0.09 *p* < 0.001	−0.00 ± 0.04 *p* = 0.7

## Data Availability

The data that support the findings of this study are available on request from the corresponding author. The data are not publicly available due to privacy or ethical restrictions.
